# Site-Specific Differences in Bone Mineral Density of Proximal Femur Correlate with the Type of Hip Fracture

**DOI:** 10.3390/diagnostics13111877

**Published:** 2023-05-27

**Authors:** Ning Li, Yi Yuan, Lu Yin, Minghui Yang, Yandong Liu, Wenshuang Zhang, Kangkang Ma, Fengyun Zhou, Zitong Cheng, Ling Wang, Xiaoguang Cheng

**Affiliations:** 1 Department of Traumatic Orthopedics, Beijing Jishuitan Hospital, Beijing 100035, China; lining_jst@126.com (N.L.); doctyang0125@126.com (M.Y.); 2 Department of Radiology, Beijing Jishuitan Hospital, Beijing 100035, China; doctoryuan@bjmu.edu.cn (Y.Y.); drliuyd@bjmu.edu.cn (Y.L.); zwsgoforit@bjmu.edu.cn (W.Z.); mkk2030@163.com (K.M.); zfy13063561620@163.com (F.Z.); 2211210490@bjmu.edu.cn (Z.C.); xiao65@263.net (X.C.); 3 Medical Research and Biometrics Center, National Center for Cardiovascular Disease, Beijing 100037, China; yinlu@mrbc-nccd.com

**Keywords:** hip fracture, femoral neck fracture, intertrochanteric fracture, bone mineral density, quantitative computed tomography

## Abstract

The aim of this study was to investigate whether site-specific differences in bone mineral density (BMD) of proximal femur correlate with the type of hip fracture using quantitative computed tomography. Femoral neck (FN) fractures were classified as nondisplaced or displaced subtypes. Intertrochanteric (IT) fractures were classified as A1, A2, or A3. The severe hip fractures were identified as displaced FN fractures or unstable IT fractures (A2 and A3). In total, 404 FN fractures (89 nondisplaced and 317 displaced) and 189 IT fractures (76 A1, 90 A2, and 23 A3) were enrolled. Areal BMD (aBMD) and volumetric BMD (vBMD) were measured in the regions of total hip (TH), trochanter (TR), FN, and IT of the contralateral unfractured femur. IT fractures exhibited lower BMD than FN fractures (all *p* ≤ 0.01). However, unstable IT fractures had higher BMD compared with stable ones (*p* < 0.01). After adjusting for covariates, higher BMD in TH and IT were associated with IT A2 (A1 vs. A2: odds ratios (ORs) from 1.47 to 1.69, all *p* < 0.01). Low bone measurements were risk factors for stable IT fractures (IT A1 vs. FN fracture subtypes: ORs from 0.40 to 0.65, all *p* < 0.01). There are substantial site-specific differences in BMD between IT fractures A1 and displaced FN fractures. Higher bone density was associated with unstable IT fracture when compared with stable ones. The understanding of biomechanics of various fracture types could help to improve the clinical management of these patients.

## 1. Introduction

Worldwide, osteoporotic hip fractures rank among the top 10 causes of disability, resulting in a high cost for the health care system and an increased risk of death in the elderly population [1,2,3]. Hip fractures are divided into two main anatomical types: femoral neck (intracapsular) fractures and intertrochanteric (extracapsular) fractures. In addition, femoral neck (FN) fractures can be further classified as nondisplaced fracture patterns (Garden Type I or II) or displaced fracture patterns (Garden Type III or IV) [4]. The AO Foundation/Orthopaedic Trauma Association (AO/OTA) classification for intertrochanteric (IT) fractures was published in 2018. The IT fractures can be further graded among three groups (A1, A2, and A3) [5]. Although several studies have regarded two main types (FN and IT) of hip fractures as a single entity, management strategies and prognosis usually differ, particularly for the severe hip fractures, such as displaced FN fractures and unstable IT fractures [6]. For example, displaced FN fractures are currently treated with arthroplasty, while IT fractures, in particular A3 type fractures, were more frequently treated with an intramedullary nail [6]. Because the hip fracture classification is the most commonly used method to determine appropriate treatment, it is worthwhile to assess the risk factors associated with subtypes of hip fractures, such as the effect on bone stabilization and the treatment outcomes. Two previous reports showed that older age, corticosteroid use, vitamin D (25[OH]D) status, and BMD are associated with severe hip fractures in women. However, the risk factors for hip fractures type and severity are still unclear [7,8].

Low bone mineral density (BMD) is an important risk factor for hip fractures [9]. Areal bone mineral density (aBMD), measured by dual energy X-ray absorptiometry (DXA), is also thought to be a possible surrogate endpoint for osteoporotic fractures [10]. Compared with DXA, quantitative computed tomography (QCT) can measure the distribution of volumetric bone mineral density (vBMD) and bone mass from three-dimensional information [11]. Previous studies reported that, compared with FN fractures, patients with IT fractures had a lower BMD and less bone mechanical strength, in particular in the trochanter region [12,13]. As the anatomic locations have different amounts and distributions of bone, the etiology and biomechanics of each fracture type may differ. However, the potential differences in bone features in the type and severity of hip fractures in older adults are currently unclear. To our knowledge, only one study [7] found that low areal BMD was related to nondisplaced FN fractures and stable IT fractures by independently comparing patients with subtypes of hip fractures with subjects without hip fractures. This study, unfortunately, did not compare the differences in BMD between different subtypes directly, and the outcomes were limited to women. The understanding of the etiology and biomechanics of various fracture types is of particular importance because a better identifying of risk factors associated with each fracture subtype and level of severity could improve the clinical management of these patients. Therefore, the aim of this study was to investigate whether site-specific differences in BMD of proximal femur correlate with the type of hip fractures in older individuals using preoperative QCT scans.

## 2. Materials and Methods

### 2.1. Participants

**Figure 1:**
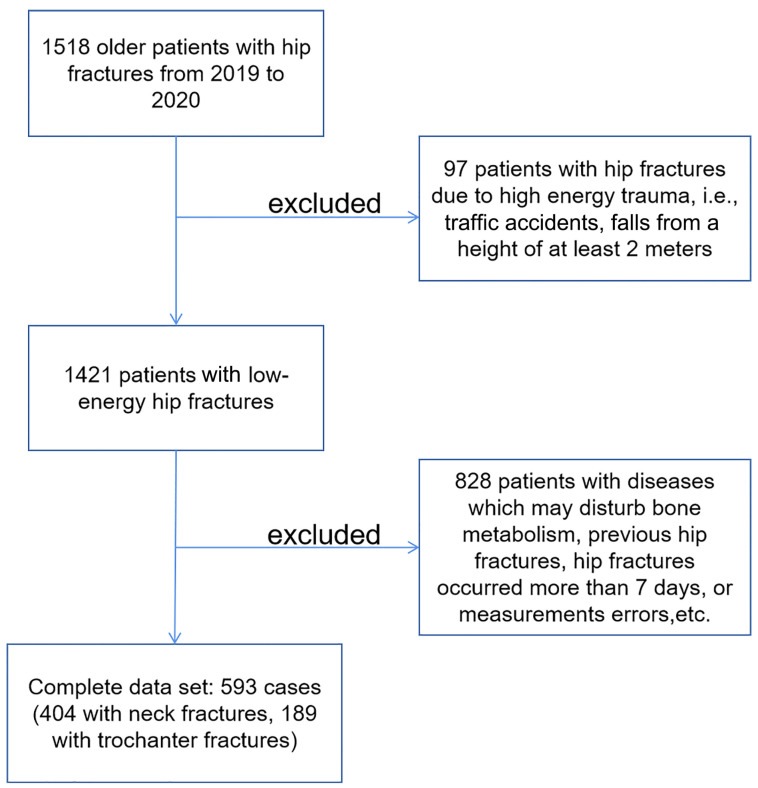
Flow chart of participant selection for the study.

This study considered a total of 1518 older subjects (aged ≥ 60 years) who had hip fractures and were admitted to the Beijing Jishuitan Hospital Emergency Department of Orthopaedic Trauma between January 2019 and December 2020; see Figure 1. CT scans were taken within 7 days after fracture to minimize changes in bone mineral density. Individuals who met the following criteria were included: hip fracture resulting from low-energy injury (limited to falls when walking or standing); Chinese Han nationality; underwent QCT scan < 48 h after injury; unilateral hip fracture; and aged ≥ 60 years. 

The exclusion criteria included: previous hip fracture(s); diseases leading to long-term limitation of activity, such as paralysis, a poorly healed lower extremity fracture, hip dysplasia, or avascular necrosis of the femoral head; painful diseases within the past 3 months, such as acute pancreatitis or lumbar fracture; metabolic bone disease (other than senile osteoporosis or postmenopausal osteoporosis); inflammatory arthritis, such as rheumatoid arthritis; indications of bone tumor or tumor-like lesion(s) of the proximal femur, such as bone metastases, chondrosarcoma, or bone island; malignant tumors with the potential to metastasize to bone; treatments that could affect the metabolism of bone tissue; and medications known to affect bone metabolism (e.g., glucocorticoids). In brief, only fully ambulatory, community-dwelling Chinese Han adults with a hip fracture resulting from low-energy injury (falls from standing or sitting height) were included. Subjects with inability to stand or walk before the event of hip fracture were excluded. 

The study was approved by the ethics committee of Beijing Jishuitan Hospital. Informed consent was obtained from each participant.

### 2.2. QCT Scans

Spiral CT imaging of the hip was performed for all study participants using one 64-slice United Imaging CT scanner (United Imaging Healthcare Co., Shanghai, China). The CT scans were acquired immediately after physical examination by orthopedic surgeons in the ER. The CT scanner was equipped with a Mindways QCT calibration phantom (Mindways Software Inc., Austin, TX, USA), which enabled the acquisition of hip CT scans according to QCT procedures. Both hips were scanned in the supine position from the top of the acetabulum to 3 cm below the lesser trochanter. The scan parameters were as follows: 120 kVp, 125 mAs, 1 mm thickness, 50 cm field of view (SFOV), and 512 × 512 matrix in spiral reconstruction and standard reconstruction.

### 2.3. CTXA Measurements

**Figure 2:**
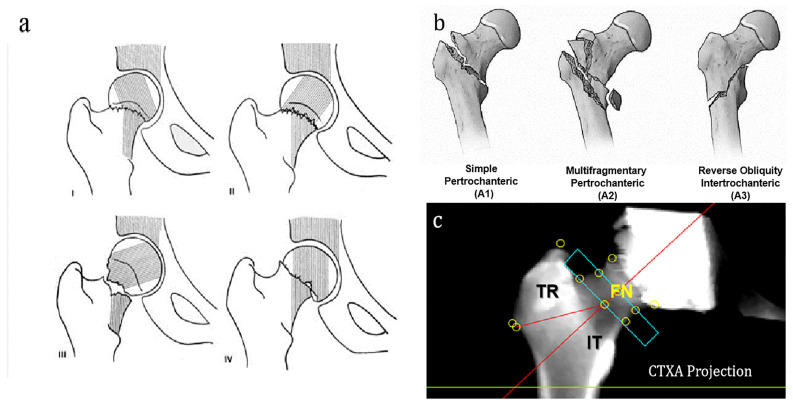
Subtypes of neck and intertrochanteric fractures and CTXA measurements. (**a**) Garden’s classification of femoral neck fracture: nondisplaced fracture patterns (Garden Type I or II) and displaced fracture patterns (Garden Type III or IV) [5]. (**b**) The AO Foundation/Orthopaedic Trauma Association (AO/OTA) classification of intertrochanteric fractures [5]. (**c**) The CTXA projection image and the regions of interest (ROIs).

Hip scan images were transferred to a QCT workstation and analyzed using the CTXA hip function version 4.2.3 of Mindways QCTPro software (Mindways Software Inc., Austin, TX, USA). The unfractured hips were chosen for analysis. A hip box was automatically placed on the hip with a crosshair approximately centered over the femoral neck as identified on the axial images. Then, the hip box automatically extracted the proximal femur by segmenting the bone from surrounding soft tissue. After image segmentation and manipulation of proximal femur rotation, a two-dimensional projection image (similar to a DXA image) was generated from a 3D CT dataset, as previously described [14]. The CTXA Hip module uses the CTXA projection images to estimate the position of volumes of interest (VOIs) commonly used in DXA hip imaging. CTXA is a multistep procedure. First, the femoral bone is separated from surrounding soft tissue using a fixed threshold with a default value of 100 mg/cm^3^. CTXA then calculates a 2D projectional image from the segmented femur [14]. Clearly, the results depend on the direction of the projection; therefore, the segmented femur must be carefully aligned manually in a predefined coordinate system. Regions of interest (ROIs) determined in the projected image are used to calculate DXA-equivalent aBMD results of the neck (FN), trochanter (TR), and intertrochanter (IT). The distal end of the IT ROI is typically adjusted to coincide with the distal end of the lesser trochanter, as seen in Figure 2. The position of the CTXA-defined FN is called a fixed location neck VOI. It can be moved by the user along the neck axis, but in the analysis presented here, only the fixed location neck VOI was used. FN, TR, and IT BMC and volume add up to total hip (TH) BMC and volume, respectively. For the measurement of a true physical BMD in mg/cm^3^ the 2D neck, trochanter and intertrochanter ROIs are projected back into the acquired 3D CT dataset to determine their 3D counterparts [15,16].

After appropriate calibration to DXA, CTXA aBMD values of the TH and FN are equivalent to DXA aBMD values and can be used to calculate T-scores according to the WHO definition of osteoporosis [17]. 

The QCTPro database was used to store and output the results for volumetric BMD (mg/cm^3^), bone mineral content (BMC, mg), and volume (cm^3^) for each of the ROIs. 

### 2.4. Subtypes of Neck and Trochanter Fractures

FN fractures were classified as nondisplaced subtypes (Garden Type I or II) and displaced subtypes (Garden Type III or IV) Figure 2. 

The AO Foundation/Orthopaedic Trauma Association (AO/OTA) fracture classification 2018 [5] was used to classify IT fractures into three groups (A1, A2, or A3) Figure 2.

On the basis of hip X-ray and CT images, all subtypes of neck and trochanter fractures were reviewed and classified by an experienced orthopedic surgeon (NL) in collaboration with an experienced musculoskeletal radiologist (YY). The severe hip fractures were referred to as displaced FN fractures (Garden III and Garden IV) or unstable IT fractures (A2 and A3).

### 2.5. Statistics

Continuous variables were described as mean ± standard deviation (SD). Mean differences between 2 groups were analyzed using the Mann–Whitney U test. The Kruskal–Wallis H test was used to compare 3 or more groups. On the basis of the results of comparisons, the odds ratios (OR) of variables for hip fracture types were calculated, adjusted for age, sex, and BMI. The ORs were normalized for a one-standard-deviation (SD) decrease in the test variable and reported with 95% confidence intervals (CIs). Statistical analysis was performed using The Statistical Analysis System (SAS 9.4 for Windows; SAS Institute Inc., Cary, NC). Differences were considered significant at *p* < 0.05. 

## 3. Results

### 3.1. Characteristics of the Study Groups

A total of 593 hip fracture patients were enrolled in the study. There were 404 FN fractures (89 nondisplaced and 317 displaced) and 189 IT fractures (76 A1, 90 A2, and 23 A3). The demographic data of the five groups grouped by classification are summarized in Table 1. There was no statistical difference of gender, age, height, weight, or BMI between nondisplaced and displaced FN fractures. IT fractures A1, A2, and A3 had significant gender and weight differences (*p* < 0.01, < 0.01, respectively). Gender, age, and weight differences between the five groups were significant (*p* < 0.01, < 0.01, 0.02, respectively). There were significant differences in age and days from injury to emergency between FN fractures and IT fractures (*p* < 0.01 and 0.03, respectively).

### 3.2. CTXA Analysis by QCT

CTXA measurements were compared among five groups, as shown in Table 1. IT fractures exhibited lower aBMD, vBMD, and bone mass than FN fractures in three areas (TH: *p* < 0.01, *p* < 0.01, *p* = 0.01; FN: *p* < 0.01, *p* < 0.01, *p* < 0.01; TR: *p* < 0.01, *p* < 0.01, *p* < 0.01, respectively), and lower IT aBMD and IT vBMD (*p* < 0.01, *p* < 0.01, respectively). Differences were not statistically significant in TH, TR, IT, or FN between nondisplaced and displaced FN fractures. aBMD and bone mass showed significant differences among three groups of IT fractures in four areas (TH: *p* < 0.01, *p* < 0.01; FN: *p* = 0.02, *p* < 0.01; TR: *p* = 0.02, *p* < 0.01; IT: *p* < 0.01, *p* < 0.01, respectively), and IT vBMD exhibited significant differences (*p* = 0.03). Femoral neck–shaft angle, bone mass, aBMD, and vBMD showed significant differences among five groups of hip fractures in TH, TR, IT, and FN (*p* ≤ 0.01). 

Based on Table 1, the ORs for hip fracture types of variables with *p* < 0.01 were identified, adjusted for age, sex, and BMI (Table 2). From Table 2, variables with *p* < 0.01 were calculated between IT fractures A1 and displaced FN fractures, including femoral neck–shaft angle, aBMD, vBMD, and bone mass in TH, TR, IT, and FN (ORs from 0.40 to 0.73). Bone mass, aBMD, and vBMD in TH and TR, FN aBMD, FN bone mass, IT aBMD, and IT vBMD were calculated (ORs from 0.47 to 0.83), with *p* < 0.01 between IT fractures A1 and nondisplaced FN fractures (Table 2). Femoral neck–shaft angle, TH aBMD, TH vBMD, FN aBMD, TR aBMD, TR vBMD, TR bone mass, and IT aBMD were calculated (ORs from 0.46 to 0.76), with *p* < 0.01 between IT fractures A2 and displaced FN fractures (Table 2). There were only four calculated parameters (femoral neck–shaft angle, TR aBMD, TR vBMD, and TR bone mass) with *p* < 0.01 between IT fractures A2 and nondisplaced FN fractures (Table 2). Femoral neck–shaft angle, TR aBMD, and TR vBMD were calculated, with *p* < 0.01 between IT fractures A3 and displaced FN fractures (not shown in Table 2). Femoral neck–shaft angle, aBMD, vBMD, and bone mass in TH, TR, IT, and FN had no significant differences between IT fractures A3 and nondisplaced FN fractures (not shown in Table 2).

On the basis of Table 1, the ORs for the variables of IT fracture types with *p* < 0.01 were identified, adjusted for age, sex, and BMI (Table 3). From Table 3, variables with *p* < 0.01 were calculated between IT fractures A2 and A1, including TH aBMD, IT aBMD, and IT vBMD. TH bone mass and IT bone mass were calculated, with *p* < 0.01 between IT fractures A3 and A1.

## 4. Discussion

In this study, we investigated hip BMD and hip bone mass differences between two main types of hip fractures and among five subtypes of hip fractures using CTXA. Investigated features included femoral neck–shaft angle, bone mass, aBMD, and vBMD in TH, TR, IT, and FN. Our results suggested that bone mass, aBMD, and vBMD in TH, TR, IT, and FN had differences between FN fractures and IT fractures and between IT fractures A1 and displaced FN fractures, whereas four areas of reduced bone mass, aBMD, and vBMD might be more relevant in IT fractures A1.

We found that subjects with IT fractures had smaller femoral neck–shaft angles, lower aBMD, lower vBMD, and lower bone mass in TH, TR, IT, and FN compared with FN fractures subjects. Femoral neck–shaft angle has been linked to altered biomechanical patterns and, thus, fracture resistance. Moreover, variations in the femoral neck–shaft angle can be used to examine the influence of loading patterns on bone quality [18]. The loading pattern was compression at the inferomedial site of the FN and tensile stresses at the superolateral site [18]. Su et al. found that IT fractures exhibited lower trabecular BMD in women and lower cortical BMD in men at the supero-posterior quadrant [19]. These findings agreed with our findings that IT fracture patients had a smaller femoral neck–shaft angle. Previous studies addressed the BMD differences between FN fractures and IT fractures. Yu et al. discovered that IT fractures had lower vBMD than FN fractures [20]. Li et al. reported that patients with IT fractures had a lower aBMD and less bone mechanical strength than those with FN fractures [12]. Taylor et al. discovered that femoral neck bone mass can predict real-time femoral neck strains and fracture loads [21]. In our study, QCT results also showed that patients with IT fractures had lower vBMD, lower aBMD, and lower bone mass compared with FN fractures. Our outcomes were comparable to these previous studies. These results suggest that BMD and bone mass may help determine the main types of hip fractures.

Techniques such as voxel-based morphometry (VBM) and surface-based statistical parametric mapping (SPM) enable local multiparametric assessments of the spatial distribution of vBMD and structural features of the proximal femur from QCT images. These assessments provide insight into how the three-dimensional (3D) variation of bone geometry, bone size, and cortical and trabecular bone compartments contribute to bone strength [20,22]. However, aBMD also captures the information of bone size, so in some cases, aBMD works well. In our study, we did not perform the complicated VBM or SPM analysis but derived the vBMD only from the commercial CTXA module. Interestingly, we found significant aBMD differences among IT fracture A1 group, IT fracture A2 group, and IT fracture A3 group. Su et al. reported that significant lower aBMD values (TH, FN, IT, and TR) were observed in the IT fractures of Chinese women and concluded that IT fractures were associated with more obvious bone loss [19]. Johannesdottir et al. [23] reported no significant vBMD differences between FN and IT fractures. Duboeuf et al. [24] found that hip aBMD is a better predictor of the IT fracture than of the FN fracture. These findings were similar to our results. Thus, these results suggest that aBMD may play a more important role than vBMD in the IT fracture.

In this study, we found that lower aBMD, lower vBMD, and lower bone mass TH, TR, IT, and FN were associated more with IT fractures A1 than with displaced FN fractures. Hey et al. reported that TR aBMD and IT aBMD were relatively denser in the FN fractures than in the IT fractures [25]. Wu et al. found that only TR aBMD was significantly lower in IT fractures than in FN fractures, and mere TR aBMD may determine hip fractures, particularly for IT fractures [26]. It is possible that the force from a fall on the lateral hip may be transferred through the relatively denser bone in the TR and IT to the less dense neck of the femur and Ward’s triangle where the fracture occurs [25]. Moreover, IT BMD decreases with age, which leads to IT fractures before the impact energy is transferred to the FN [27]. In addition, low BMD might protect against FN fractures if fractures of the IT region dissipate the energy of a direct impact on the hip [28]. Furthermore, Cauley et al. found that each SD decrease in FN BMD was associated with a >3-fold increased risk of nondisplaced FN fractures, and lower FN BMD was associated with a higher risk of stable IT fractures than of unstable IT fractures [7]. Thus, the previous study concluded that the lower the BMD, the greater the likelihood of experiencing a hip fracture that is less displaced and more stable [7]. However, this study failed to show an association between nondisplaced femoral and stable IT fractures. Lower FN BMD was found to be more strongly associated with IT fractures A1 than displaced or nondisplaced FN fractures in our study. A possible explanation could be that other skeletal factors may also determine the severity and type of hip fractures, for example, hip axis length and FN diameter [13]. Furthermore, the diameter of IT is twice that of the FN, the area moment of inertia of the IT is 8 times superior to the FN, and the polar moment of inertia of the IT is 16 times greater than that of the FN [26]. According to biomechanics, FN fractures should occur more frequently. These results may indicate that hip fracture patterns may be influenced by the pattern of reduced BMD in different areas of the hip.

Interestingly, when we calculated the ORs of the variables of hip fracture type, there were no differences in aBMD, vBMD, or bone mass in TH, TR, IT, and FN between IT fractures graded A3 and nondisplaced FN fractures. The most common fall pattern in the elderly is lateral impact on the trochanter [29]. Rudman et al. demonstrated that the femoral neck sustains compressive loads in a one- or two-legged stance, while the trochanter still sustains tensile loads [30]. They developed a 2D model that showed that, in principle, including ligamentous and muscular forces had the effect of generating compressive stresses across most of the proximal femur [30]. Meanwhile, the energy of a direct impact to the hip is dissipated when transmitted from the IT to the FN [8]. In addition, our study indicated that lower BMC and BMD in TH and IT were associated with IT fractures A1 rather than with A2 or A3. IT fractures A1 occur at the translational area of the cervico-trochanteric junction. Low BMD, according to Szuic et al., is associated with an increased risk of hip fractures, particularly IT fractures [31]. The IT BMD drops severely in keeping with Wolff’s law and may introduce IT fractures [26]. Hence, the incidence of IT fractures may be determined by BMD, but the incidence of FN fractures may be more likely to be dependent on multiple factors. Nevertheless, further studies are needed to confirm this conclusion.

This study had several limitations. First, rather than predicting hip fractures, this study evaluated associations of different types of hip fractures. Second, our study lacked a baseline QCT scan prior to the occurrence of the FN and IT fractures. Third, all subjects were Chinese or Asian people, limiting the interpretation of the results to other ethnicities. Last, we did not include comparisons with more geometric parameters but focused mainly on bone density and mass.

## 5. Conclusions

In conclusion, we found substantial site-specific differences in BMD between IT fractures A1 and displaced FN fractures throughout the proximal femur. Higher bone density was associated with unstable IT fracture when compared with stable ones. The understanding of biomechanics of various fracture types may help improve the clinical management of these patients.

## Figures and Tables

**Table 1:** General characteristics by fracture types.

Characteristics (Mean ± SD)	Total (*n* = 593)	Nondisplaced FN Fracture (*n* = 87)	Displaced FN Fracture (*n* = 317)	Total FN Fracture (*n* = 404)	*p*-Value ^1^	IT Fracture A1 (*n* = 76)	IT Fracture A2 (*n* = 90)	IT Fracture A3 (*n* = 23)	Total IT Fracture (*n* = 189)	*p*-Value ^2^	*p*-Value ^3^	*p*-Value ^4^
Male, % (*n*)	27.99 (166)	24.14 (21)	27.76 (88)	26.98 (109)	0.50	22.37 (17)	27.78 (25)	65.22 (15)	30.16 (57)	<0.01	<0.01	0.42
Age (years)	79.59 ± 7.62	77.67 ± 7.53	78.68 ± 7.70	78.46 ± 7.66	0.25	81.57 ± 6.84	82.55 ± 7.12	81.18 ± 6.80	81.99 ± 6.95	0.53	<0.01	<0.01
Height (cm)	161.50 ± 8.22	161.66 ± 7.86	161.89 ± 7.44	161.84 ± 7.52	0.71	160.08 ± 7.25	161.16 ± 6.93	161.57 ± 19.68	160.78 ± 9.54	0.06	0.15	0.34
Weight (kg)	60.68 ± 11.36	60.61 ± 11.93	60.86 ± 10.73	60.81 ± 10.99	0.71	57.58 ± 10.87	60.94 ± 12.85	67.26 ± 10.55	60.39 ± 12.14	<0.01	0.02	0.73
BMI (kg/m^2^)	23.33 ± 5.44	23.18 ± 4.27	23.19 ± 3.58	23.19 ± 3.73	0.57	22.42 ± 3.67	23.38 ± 4.41	28.52 ± 19.26	23.65 ± 7.95	0.05	0.17	0.93
Days from injury to emergency	1.12 ± 1.49	1.25 ± 1.36	1.21 ± 1.59	1.22 ± 1.54	0.09	1.05 ± 1.55	0.67 ± 0.90	1.35 ± 2.05	0.91 ± 1.37	0.51	0.06	0.03
Femoral neck–shaft angle	45.86 ± 6.02	46.20 ± 5.57	46.69 ± 5.49	46.58 ± 5.51	0.69	44.57 ± 7.07	44.28 ± 6.71	43.74 ± 6.08	44.33 ± 6.75	0.74	<0.01	<0.01
TH aBMD	0.63 ± 0.16	0.64 ± 0.14	0.66 ± 0.16	0.66 ± 0.16	0.62	0.54 ± 0.13	0.59 ± 0.13	0.67 ± 0.17	0.58 ± 0.14	<0.01	<0.01	<0.01
TH vBMD	223.56 ± 60.92	228.02 ± 58.09	232.52 ± 62.08	231.56 ± 61.20	0.66	194.25 ± 53.29	210.44 ± 54.23	231.18 ± 69.05	206.45 ± 56.80	0.05	<0.01	<0.01
TH bone mass	22.03 ± 9.26	22.17 ± 8.72	22.92 ± 9.94	22.76 ± 9.68	0.75	18.48 ± 7.72	20.57 ± 7.24	26.61 ± 9.64	20.46 ± 8.10	<0.01	<0.01	0.01
FN aBMD	0.54 ± 0.14	0.57 ± 0.14	0.55 ± 0.14	0.55 ± 0.14	0.35	0.48 ± 0.13	0.50 ± 0.13	0.54 ± 0.16	0.50 ± 0.14	0.02	<0.01	<0.01
FN vBMD	202.03 ± 48.01	204.68 ± 42.81	206.87 ± 46.46	206.40 ± 45.66	0.91	185.51 ± 44.57	197.2 ± 52.34	198.82 ± 67.71	192.70 ± 51.59	0.32	0.01	<0.01
FN bone mass	1.86 ± 0.54	1.97 ± 0.60	1.89 ± 0.53	1.91 ± 0.55	0.33	1.65 ± 0.52	1.76 ± 0.51	2.01 ± 0.54	1.74 ± 0.53	<0.01	<0.01	<0.01
TR aBMD	0.43 ± 0.12	0.44 ± 0.11	0.45 ± 0.12	0.45 ± 0.12	0.54	0.36 ± 0.10	0.38 ± 0.10	0.44 ± 0.14	0.38 ± 0.11	0.02	<0.01	<0.01
TR vBMD	140.88 ± 37.15	145.96 ± 33.85	148.05 ± 37.35	147.60 ± 36.59	0.76	121.15 ± 32.95	127.67 ± 32.14	139.62 ± 43.01	126.50 ± 34.23	0.11	<0.01	<0.01
TR bone mass	4.56 ± 1.77	4.85 ± 1.80	4.76 ± 1.73	4.78 ± 1.74	0.77	3.77 ± 1.80	4.10 ± 1.52	5.26 ± 1.90	4.11 ± 1.74	<0.01	<0.01	<0.01
IT aBMD	0.76 ± 0.19	0.77 ± 0.16	0.79 ± 0.19	0.79 ± 0.18	0.54	0.65 ± 0.18	0.72 ± 0.16	0.82 ± 0.20	0.70 ± 0.18	<0.01	<0.01	<0.01
IT vBMD	272.39 ± 78.07	278.41 ± 74.72	282.76 ± 79.12	281.82 ± 78.12	0.75	235.33 ± 72.76	258.25 ± 69.48	284.52 ± 85.28	252.23 ± 74.22	0.03	<0.01	<0.01
IT bone mass	15.35 ± 8.44	15.00 ± 8.23	16.10 ± 8.92	15.86 ± 8.78	0.36	12.62 ± 7.31	14.32 ± 6.99	19.30 ± 8.64	14.24 ± 7.57	<0.01	<0.01	0.06

Note: SD, standard deviation; FN fracture, femoral neck fracture; IT fracture, intertrochanteric fracture; BMI, body mass index; ^1^ *p*-value was used to compare the difference between nondisplaced FN fracture group and displaced FN fracture group; ^2^ *p*-value was used to compare the difference among IT fracture A1 group, IT fracture A2 group, and IT fracture A3 group; ^3^ *p*-value was used to compare the difference among nondisplaced FN fracture group, displaced FN fracture group, IT fracture A1 group, IT fracture A2 group, and IT fracture A3 group; ^4^ *p*-value was used to compare the difference between total FN fracture group and total IT fracture group.

**Table 2:** Odds ratios and 95% confidence intervals for fracture risk by various fracture subtypes per sex-specific SD increase in CTXA measurements.

Muscle Parameters	Unadjusted Models		Adjusted Models ^1^	
	OR (95% CI)	*p*-Value	OR (95% CI)	*p*-Value
IT fracture A1 vs. displaced FN fracture				
Femoral neck–shaft angle	0.68 (0.52, 0.89)	<0.01	0.73 (0.55, 0.96)	0.03
TH aBMD	0.43 (0.32, 0.58)	<0.01	0.42 (0.29, 0.59)	<0.01
TH vBMD	0.49 (0.36, 0.66)	<0.01	0.50 (0.36, 0.70)	<0.01
TH bone mass	0.53 (0.38, 0.75)	<0.01	0.57 (0.40, 0.81)	<0.01
FN aBMD	0.53 (0.39, 0.72)	<0.01	0.47 (0.32, 0.69)	<0.01
FN vBMD	0.60 (0.46, 0.79)	<0.01	0.65 (0.48, 0.87)	<0.01
FN bone mass	0.62 (0.48, 0.82)	<0.01	0.50 (0.34, 0.73)	<0.01
TR aBMD	0.41 (0.30, 0.56)	<0.01	0.40 (0.28, 0.57)	<0.01
TR vBMD	0.44 (0.32, 0.59)	<0.01	0.43 (0.31, 0.61)	<0.01
TR bone mass	0.51 (0.38, 0.69)	<0.01	0.46 (0.32, 0.66)	<0.01
IT aBMD	0.46 (0.34, 0.60)	<0.01	0.44 (0.32, 0.61)	<0.01
IT vBMD	0.51 (0.38, 0.68)	<0.01	0.52 (0.38, 0.72)	<0.01
IT bone mass	0.60 (0.43, 0.83)	<0.01	0.65 (0.47, 0.90)	0.01
IT fracture A1 vs. nondisplaced FN fracture				
Femoral neck–shaft angle	0.78 (0.57, 1.05)	0.10	0.83 (0.60, 1.15)	0.26
TH aBMD	0.43 (0.29, 0.63)	<0.01	0.48 (0.32, 0.72)	<0.01
TH vBMD	0.51 (0.36, 0.74)	<0.01	0.57 (0.39, 0.83)	<0.01
TH bone mass	0.60 (0.42, 0.86)	0.01	0.64 (0.44, 0.94)	0.02
FN aBMD	0.51 (0.35, 0.72)	<0.01	0.52 (0.34, 0.78)	<0.01
FN vBMD	0.61 (0.43, 0.87)	0.01	0.72 (0.49, 1.05)	0.09
FN bone mass	0.59 (0.44, 0.81)	<0.01	0.53 (0.35, 0.80)	<0.01
TR aBMD	0.43 (0.30, 0.63)	<0.01	0.48 (0.32, 0.72)	<0.01
TR vBMD	0.45 (0.31, 0.65)	<0.01	0.49 (0.33, 0.73)	<0.01
TR bone mass	0.53 (0.37, 0.75)	<0.01	0.50 (0.34, 0.74)	<0.01
IT aBMD	0.44 (0.30, 0.64)	<0.01	0.47 (0.32, 0.71)	<0.01
IT vBMD	0.53 (0.37, 0.76)	<0.01	0.57 (0.39, 0.83)	<0.01
IT bone mass	0.72 (0.51, 1.00)	0.05	0.75 (0.53, 1.06)	0.11
IT fracture A2 vs. displaced FN fracture				
Femoral neck–shaft angle	0.64 (0.49, 0.83)	<0.01	0.63 (0.48, 0.82)	<0.01
TH aBMD	0.62 (0.48, 0.80)	<0.01	0.60 (0.44, 0.80)	<0.01
TH vBMD	0.68 (0.53, 0.88)	<0.01	0.68 (0.51, 0.90)	0.01
TH bone mass	0.74 (0.56, 0.97)	0.03	0.75 (0.56, 1.01)	0.06
FN aBMD	0.73 (0.57, 0.93)	0.01	0.68 (0.50, 0.93)	0.01
FN vBMD	0.82 (0.65, 1.04)	0.10	0.86 (0.67, 1.11)	0.24
FN bone mass	0.82 (0.66, 1.02)	0.07	0.76 (0.56, 1.02)	0.07
TR aBMD	0.48 (0.36, 0.64)	<0.01	0.46 (0.33, 0.64)	<0.01
TR vBMD	0.54 (0.41, 0.71)	<0.01	0.53 (0.39, 0.72)	<0.01
TR bone mass	0.62 (0.47, 0.82)	<0.01	0.63 (0.46, 0.86)	<0.01
IT aBMD	0.66 (0.51, 0.85)	<0.01	0.65 (0.49, 0.87)	<0.01
IT fracture A2 vs. nondisplaced FN fracture				
Femoral neck–shaft angle	0.74 (0.55, 1.00)	0.05	0.71 (0.51, 0.98)	0.04
TH aBMD	0.63 (0.45, 0.89)	0.01	0.73 (0.51, 1.05)	0.09
TH vBMD	0.72 (0.52, 1.00)	0.05	0.79 (0.56, 1.11)	0.18
TH bone mass	0.77 (0.55, 1.07)	0.12	0.83 (0.59, 1.18)	0.30
FN aBMD	0.67 (0.50, 0.91)	0.01	0.75 (0.52, 1.07)	0.11
FN vBMD	0.87 (0.64, 1.17)	0.34	1.01 (0.73, 1.40)	0.95
FN bone mass	0.77 (0.59, 1.00)	0.05	0.78 (0.55, 1.09)	0.14
TR aBMD	0.49 (0.34, 0.71)	<0.01	0.56 (0.38, 0.84)	0.01
TR vBMD	0.55 (0.39, 0.78)	<0.01	0.62 (0.43, 0.90)	0.01
TR bone mass	0.59 (0.42, 0.83)	<0.01	0.64 (0.44, 0.93)	0.02
IT aBMD	0.67 (0.48, 0.94)	0.02	0.75 (0.52, 1.08)	0.12

^1^ Adjusted for age, sex, and BMI.

**Table 3:** Odds ratios and 95% confidence intervals for fracture risk by various trochanter fracture types per sex-specific SD increase in CTXA measurements.

Muscle Parameters	Unadjusted Models		Adjusted Models ^1^	
	OR (95% CI)	*p*-Value	OR (95% CI)	*p*-Value
IT fracture A2 vs. IT fracture A1				
Femoral neck–shaft angle	0.97 (0.74, 1.27)	0.82	0.92 (0.69, 1.24)	0.60
TH aBMD	1.50 (1.05, 2.13)	0.03	1.59 (1.05, 2.40)	0.03
TH vBMD	1.42 (1.00, 2.02)	0.05	1.47 (1.00, 2.16)	0.05
TH bone mass	1.40 (0.95, 2.05)	0.09	1.39 (0.93, 2.08)	0.10
FN aBMD	1.28 (0.93, 1.75)	0.13	1.39 (0.94, 2.07)	0.10
FN vBMD	1.29 (0.95, 1.76)	0.10	1.32 (0.94, 1.84)	0.11
FN bone mass	1.25 (0.94, 1.66)	0.13	1.39 (0.95, 2.05)	0.09
TR aBMD	1.26 (0.87, 1.81)	0.22	1.26 (0.83, 1.92)	0.27
TR vBMD	1.26 (0.89, 1.79)	0.19	1.32 (0.89, 1.97)	0.17
TR bone mass	1.22 (0.90, 1.66)	0.21	1.29 (0.91, 1.81)	0.15
IT aBMD	1.53 (1.09, 2.14)	0.01	1.64 (1.11, 2.41)	0.01
IT vBMD	1.44 (1.02, 2.03)	0.04	1.49 (1.03, 2.17)	0.04
IT bone mass	1.31 (0.91, 1.88)	0.14	1.26 (0.87, 1.83)	0.23
IT fracture A3 vs. IT fracture A1				
Femoral neck–shaft angle	0.94 (0.62, 1.41)	0.75	0.92 (0.54, 1.58)	0.77
TH aBMD	2.39 (1.40, 4.10)	<0.01	1.73 (0.96, 3.14)	0.07
TH vBMD	2.06 (1.25, 3.40)	<0.01	1.65 (0.94, 2.92)	0.08
TH bone mass	2.18 (1.30, 3.64)	<0.01	1.88 (1.03, 3.42)	0.04
FN aBMD	1.80 (1.17, 2.74)	0.01	1.37 (0.82, 2.30)	0.23
FN vBMD	1.42 (0.93, 2.18)	0.11	1.17 (0.71, 1.94)	0.53
FN bone mass	2.11 (1.39, 3.19)	<0.01	1.70 (1.00, 2.89)	0.05
TR aBMD	1.82 (1.12, 2.97)	0.02	1.17 (0.66, 2.08)	0.59
TR vBMD	1.73 (1.07, 2.80)	0.02	1.21 (0.69, 2.13)	0.51
TR bone mass	1.78 (1.15, 2.78)	0.01	1.24 (0.73, 2.11)	0.42
IT aBMD	2.44 (1.41, 4.22)	<0.01	1.80 (0.98, 3.30)	0.06
IT vBMD	2.01 (1.22, 3.31)	0.01	1.68 (0.95, 2.98)	0.08
IT bone mass	1.98 (1.21, 3.23)	0.01	1.86 (1.03, 3.36)	0.04

^1^ Adjusted for age, sex, and BMI.

## Data Availability

The data presented in this study are available on request from the corresponding author.
